# Adipose tissue and adipose-derived stromal cells can reduce skin contraction in an *in vitro* tissue engineered full thickness skin model

**DOI:** 10.1080/21623945.2025.2473367

**Published:** 2025-03-19

**Authors:** Victoria L. Workman, Anna‑Victoria Giblin, Nicola H. Green, Sheila MacNeil, Vanessa Hearnden

**Affiliations:** a Department of Materials Science and Engineering, University of Sheffield, Sheffield, UK; b Department of Plastic Surgery, Sheffield Teaching Hospitals, NHS Foundation Trust, Sheffield, UK; c INSIGNEO Institute, University of Sheffield, Sheffield, UK

**Keywords:** Skin contraction, adipose tissue, fat grafting, adipose-derived stromal cells, contracture

## Abstract

Skin contracts during wound healing to facilitate wound closure. In some patients, skin contraction can lead to the formation of skin contractures that limit movement, impair function, and significantly impact well-being. Current treatment options for skin contractures are burdensome for patients, and there is a high risk of recurrence. Autologous fat grafting can improve the structure and function of scarred skin; however, relatively little is known about the effect of fat on skin contraction. In this study, an in vitro tissue-engineered model of human skin was used to test the effects of adipose tissue and adipose-derived stromal cells on skin contraction. Untreated tissue-engineered skin contracted to approximately 60% of the original area over 14 days in culture. The addition of adipose tissue reduced this contraction by 50%. Adipose tissue, which was emulsified or concentrated and high doses of adipose-derived stromal cells (ADSC) were able to inhibit contraction to a similar degree; however, lower doses of ADSC did not show the same effect. In conclusion, the subcutaneous application of adipose tissue has the potential to inhibit skin contraction. This study provides in vitro evidence to support the use of autologous fat grafting to prevent skin contraction in patients most at risk.

## Introduction

An inherent feature of physiological wound healing is skin contraction that drives wound closure. Contraction commences approximately 4 to 5 days after wounding at an average speed of 0.6–0.7 mm per day, depending on tissue type and wound shape [1]. Skin contraction is initiated in the proliferative phase of wound healing as fibroblasts differentiate into myofibroblasts. Myofibroblasts, which express contractile alpha smooth muscle actin protein, act to pull the wound edges together while keratinocytes migrate from the wound edge, creating a purse string effect. In human skin, where the skin cannot glide freely over underlying tissues, this contraction leads to discomfort and, in areas over joints, restrictions in the range of motion [2]. Skin contraction is just one aspect of scar formation and does not necessarily lead to pathological scarring; however, Yannas et al. argued that skin contraction and regeneration are mutually antagonistic processes [3]. Skin contractures that limit movement are observed in approximately 33% of adult burn survivors [2] and are often worse in paediatric patient populations [4].

Current treatments for contracted skin include non-surgical solutions (such as pressure garments and splints) or surgical correction [5]. Surgical corrections such as skin grafts, z-plasty, adjacent tissue rearrangement, and flap surgery are often used in combination with postoperative physical and occupational therapy. These current treatments have a high risk of recurrence and the impact of skin contraction on a patient’s independence, mental health, and well-being can be devastating. Sheckter et al., showed that there was no improvement in patient-reported physical outcomes in patients receiving contracture surgery compared to non-surgical groups, and that patient-reported mental outcomes were lower in the surgical group, illustrating the need for novel treatment options [6].

To increase our knowledge of skin contraction and develop novel treatment strategies, a physiologically relevant skin contraction model is needed. The skin of most animals used for *in vivo* experiments contains a subcutaneous muscle layer that is not present in human skin. This layer enables the skin to glide over the underlying tissues, making these unsuitable to study human skin contraction. Many *in vitro* studies rely on the use of tethered or untethered collagen gels, and these studies have provided valuable insights into how fibroblasts contribute to wound closure, but these gels do not contain the mature cross-linked collagen found in native human dermis and cannot be directly translated to the *in vivo* situation [4,7].

In response to this need, our group has developed an *in vitro* tissue-engineered model of skin contraction using decellularised human dermis seeded with human keratinocytes and fibroblasts [8]. This model was validated using pharmacological modulators of collagen synthesis and degradation remodelling. Further, specific cross-linking pathways responsible for the contraction were identified [8]. Using this model, we were able to demonstrate that both fibroblasts and keratinocytes were needed to contract human dermis [9]. When delivered to patients, this tissue-engineered skin was shown to contract in a similar way to split-thickness skin grafts, further demonstrating the validity of this model [10].

Autologous fat grafting (AFG), in which lipoaspirate is collected and reinjected into the same patient, became popular following the development of the Coleman technique in the 1980s [11]. Initially developed to restore tissue volume following surgery or trauma, AFG has also been shown to improve the quality and elasticity of the overlying scarred skin [12,13]. Factors secreted from adipose tissue have been shown to inhibit myofibroblast differentiation [14]. There is now interest in the development of AFG for scar treatment, and evidence is emerging demonstrating the potential of AFG for skin contracture prevention and treatment [15,16].

A process known as ‘Rigottomy’, where adipose tissue is grafted into nicks made in contracted bands of tissue has been reported to improve function in patients with various contractures [15]. The evidence from these case reports is beginning to be confirmed in randomized controlled trials. For example, the study by Abouzaid *et al*. showed a significant reduction in contractures in patients who received AFG plus a nanofat dressing in comparison to routine treatment with dressings [16].

To fully utilize and exploit the benefits of AFG for scar contractures, further evidence is needed to understand the underlying mechanisms. The ability of adipose tissue to reduce skin contraction has not been previously investigated in laboratory studies; however, this understanding is critical to provide the evidence needed to support future clinical studies in this promising area of research. The aim of this study was to investigate how the addition of adipose tissue and adipose-derived stromal cells (ADSC) affected skin contraction in an *in vitro* model.

## Results

**Figure 1.**
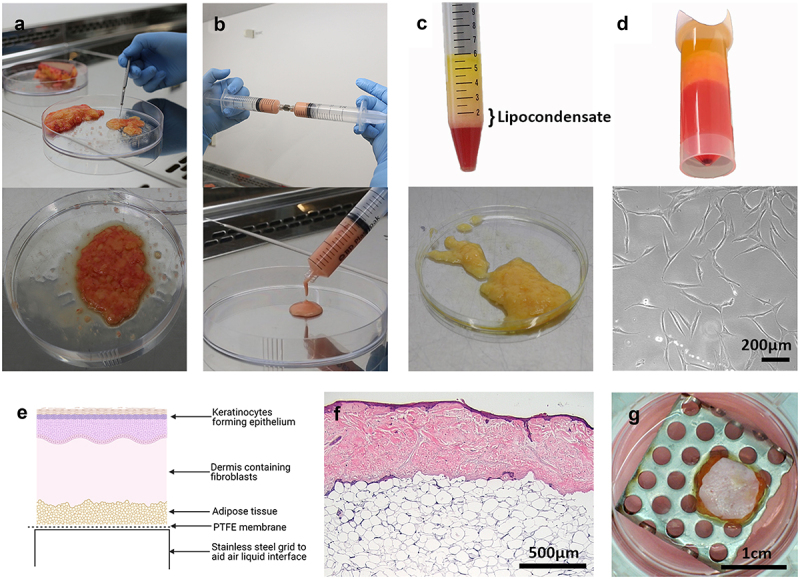
Processing and preparation of adipose tissue and adipose-derived stromal cells. (a) Excised adipose tissue was minced to a consistency which resembled lipoaspirate to facilitate addition of adipose tissue to the tissue-engineered skin model. (b) Adipose tissue was emulsified by passing between two Luer-Lok syringes 30 times to generate emulsified fat. (c) Emulsified fat was filtered and centrifuged and multiple layers formed including: free lipid (top layer), lipocondensate (mid layer) and red aqueous component (bottom layer). The lipocondensate layer was separated from the other layers and added to the tissue engineered skin model. (d) Minced adipose tissue was incubated with collagenase to isolate adipose-derived stromal cells (ADSC) which were subsequently expanded through adherent culture. Lower image shows appearance of ADSC in culture. (e) Schematic of tissue-engineering skin showing the location of PTFE membrane, above which adipose tissue or ADSC were added. (f) H&E stained section of tissue engineered skin with adipose tissue added after 14 days of culture at air-liquid interface. Expected layers are present including a stratified epithelium comprised of keratinocytes, dermis populated with fibroblasts and hypodermal layer made up of adipocytes and other adipose cells. (g) Aerial photograph of tissue engineered skin model with adipose tissue underneath.

Figure 1 illustrates how adipose tissue was processed before it was added to the tissue-engineered skin model. Adipose tissue obtained from routine plastic surgery was minced following excision to a consistency that resembled that of lipoaspirate (Figure 1a). Further processing was conducted to produce emulsified fat (Figure 1b) and lipocondensate (Figure 1c). ADSC were isolated by collagenase digestion and adherence culture (Figure 1d) to study the effects of different adipose tissue components on skin graft contraction. Adipose tissue or ADSC were placed beneath the fibroblast-populated dermis (Figure 1e). The resulting histological and macroscopic appearances of the tissue-engineered skin models are shown in Figure 1f and **g**, further details of which can be found in our recent publication [17].

**Figure 2.**
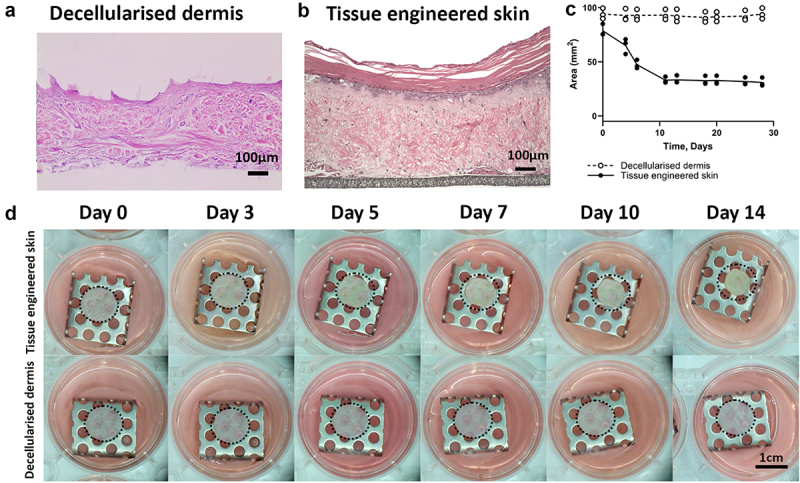
Tissue-engineering skin model of contraction. (a) DED stained with H&E shows a mature dense irregular collagen matrix. (b) Histological analysis of the tissue-engineering skin on day 14 showing a stratified epithelium on top of a fibroblast populated dermis with the PTFE support membrane visible at the bottom. (c) The area (measured in mm^2^) covered by the models was measured at each time point. Open circles show the surface area of DED did not change over 28 days in culture. Tissue-engineering skin models (black circles) decreased in area over time and reached a plateau around 10 days, which was sustained until day 28. Data from three models per condition were plotted separately and mean values are shown by the dotted line (decellularised dermis) or solid line (tissue-engineering skin model). (d) Aerial images of tissue-engineering skin and decellularised dermis (DED) over 14 days of culture at air-liquid interface. Dotted outline shows the size of the model at day 0. DED did not decrease in size over time. Tssue-engineering skin (comprised of fibroblasts and keratinocytes seeded onto DED) contracted over 14 days in culture.

Tissue-engineered skin models consisting of decellularised dermis (Figure 2a) seeded with primary human fibroblasts and primary human keratinocytes were cultured submerged for 48 h before being raised to an air – liquid interface (ALI), as previously described [8]. In this study, ‘day 0’ refers to when the skin model was raised to ALI and adipose tissue or ADSC were added. Once raised to ALI, the adhered keratinocytes stratified, forming a multilayer squamous epithelium (Figure 2b), and the tissue engineered model contracted (Figure 2c,d). The degree of contraction was determined by measuring the change in the surface area from aerial photographs of the models in culture (Figure 2d). Tissue-engineered skin models without adipose tissue contracted between days 0 and 14, with the most rapid period of contraction between days 4 and 7 (Figure 2c). The models contracted to approximately 60% of the original area after 14 days of culture at the air-liquid interface (ALI), after which contraction plateaued (Figure 2c).

**Figure 3.**
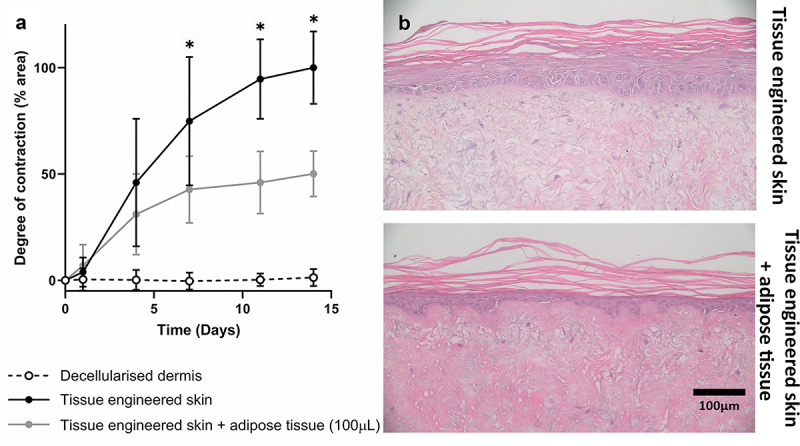
Addition of adipose tissue reduces contraction in a tissue-engineering skin model and promotes a more physiological dermal epidermal junction. (a) Graph to show the degree of contraction relative to tissue-engineering skin control. Decellularised dermis did not contract (0% contraction) while tissue-engineering skin models (DED with keratinocytes and fibroblasts) steadily contracted over 14 days (presented as 100% contraction at day 14). The addition of 100 µl of adipose tissue reduced contraction significantly at days 7, 11 and 14 compared to tissue-engineering skin alone (*p < 0.0001) *n* = 9. Mean ± S.D. (b) H&E stained histology sections showing the appearance of tissue-engineering skin with and without adipose tissue. Models cultured without adipose tissue (upper image) had a flat dermal epidermal junction and multi-layered epidermis. In comparison, tissue-engineering skin cultured with the addition of adipose tissue (lower image) had a thinner epidermis with more rete ridge like projections.

Minced adipose tissue (100 µL) was added beneath the tissue-engineered skin models and the degree of contraction was measured over 14 days (Figure 3a). The decellularised dermis (DED) without any cells did not contract over 14 days; however, tissue-engineered skin models contracted rapidly until day 7. After day 7, the contraction slowed until it plateaued at day 14 (defined as 100% contraction). The addition of adipose tissue on day 0 slowed the rate of contraction and led to a significantly lower degree of contraction compared with the tissue-engineered skin model at days 7, 11 and 14. On day 14, models treated with adipose tissue had contracted 50% less than tissue-engineered skin models without adipose treatment.

Histological analysis showed that a stratified squamous epithelium formed in the contracted skin model (with and without adipose tissue) with a keratinized superficial layer (Figure 3b). Tissue engineered skin models with adipose tissue had a dermal epidermal junction that more closely resembled native skin compared to models without adipose tissue, and an increase in rete ridge-like projections was observed, as previously reported [17].

**Figure 4.**
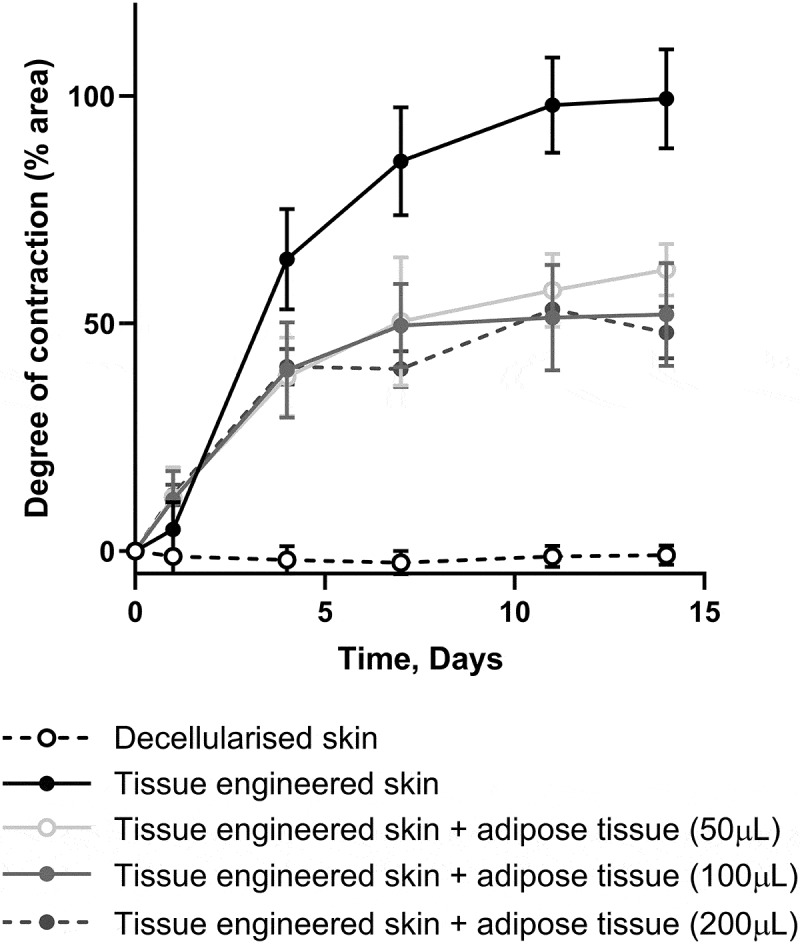
The addition of different amounts of adipose tissue did not affect the degree of contraction. Graph showing the degree of contraction of tissue-engineering skin models treated with 50, 100, or 200 µl of adipose tissue compared to models without adipose tissue. All doses of adipose tissue significantly decreased the amount of contraction compared to tissue-engineering skin models (*p* < 0.001) from day 7 onwards. There was no significant difference in the degree of contraction between the different doses of AT. *n* = 6, except for 200 µl adipose tissue, where *n* = 3. Mean ± S.D.

We next investigated how the dose of adipose tissue (50, 100, or 200 µL) affected skin contraction (Figure 4). Irrespective of the amount of adipose tissue added, the observed inhibition of contraction was similar. There was no difference in the degree or rate of contraction when different amounts of adipose tissue were added to the models.

**Figure 5.**
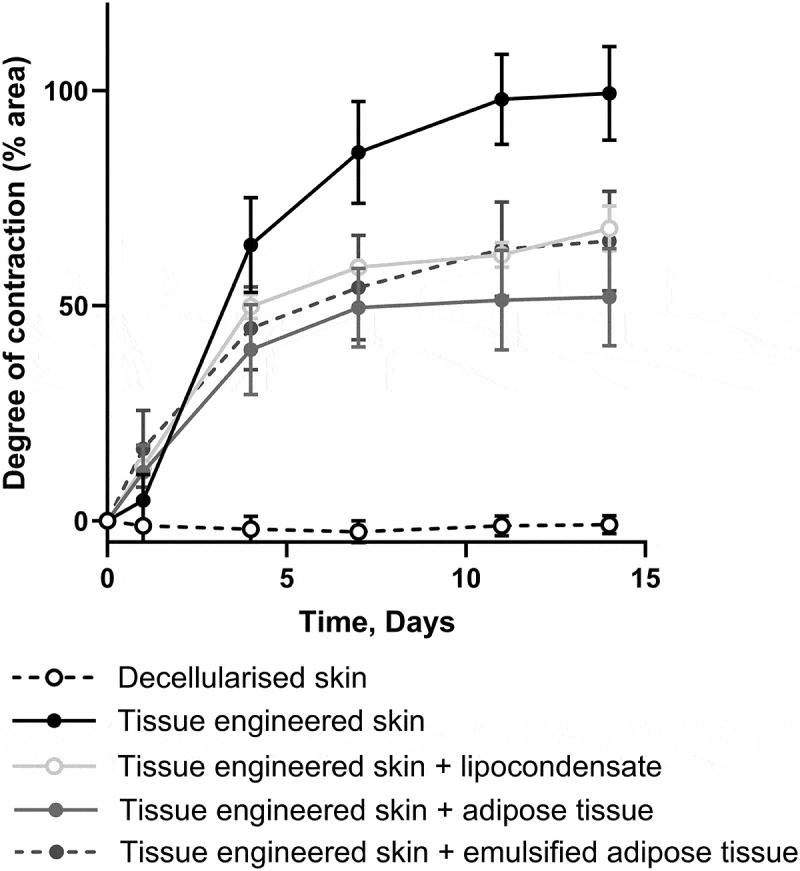
Processing adipose tissue did not change the amount of contraction. The graph shows the degree of contraction of tissue-engineering skin models treated with 100 µl of adipose tissue, lipocondensate, or emulsified adipose tissue compared to the tissue-engineering skin model without adipose tissue. All formulations of adipose tissue significantly decreased the amount of contraction (*p* < 0.001) compared to tissue-engineering skin models without adipose tissue from day 7 onwards. There was no significant difference in the degree of contraction between the adipose tissue formulations. *n* = 6, except for lipocondensate, for which *n* = 2. Mean ± S.D.

Adipose tissue processing has been investigated clinically to alter the composition of adipose tissue and further optimize adipose derived therapies. Here, we tested how adipose tissue processing, to emulsify tissues or condense cells, affected its ability to inhibit skin contraction when compared to minced adipose tissue (Figure 5). The addition of processed adipose tissue resulted in the same reduction in skin contraction as that of unprocessed adipose tissue.

**Figure 6.**
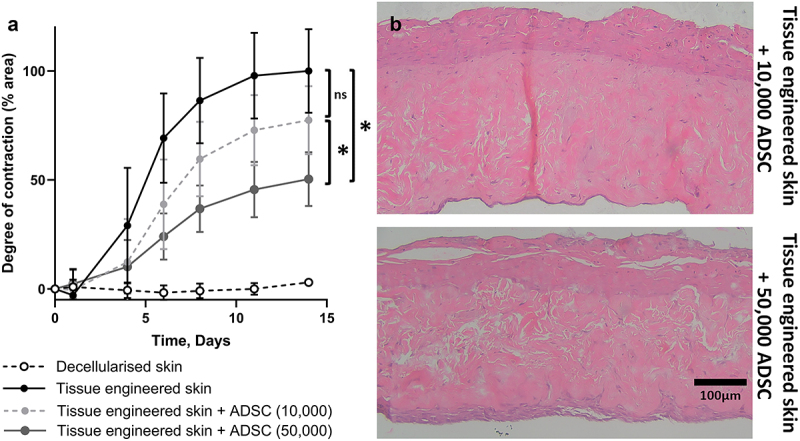
Addition of ADSC to tissue-engineering skin inhibited contraction. (a) Graph to show the degree of contraction of tissue-engineering skin models treated with 50,000 or 10,000 ADSC compared to tissue-engineering skin model without adipose cells. The addition of 50,000 ADSC significantly reduced contraction (*p* > 0.001) compared to tissue-engineering skin models without adipose cells from day 6 onwards. The addition of 10,000 ADSC significantly reduced contraction compared to tissue-engineering skin models at days 6, 8 and 11 (*p* < 0.05) but this difference was non-significant (ns) at day 14. There was a significant difference in contraction between the two ADSC doses at days 8, 11 and 14 (*p* < 0.05). *n* = 8. Mean ± S.D. (b) Representative images showing histological appearance of tissue-engineering skin models cultured with ADSC for 14 days. Epithelium can be observed at the top of the image and ADSC on the bottom surface of the fibroblasts populated DED.

The effect of adipose-derived stromal cells (ADSC) was investigated by adding 10,000 or 50,000 cells beneath tissue-engineered skin (Figure 6). The addition of ADSC led to a decrease in contraction compared to tissue-engineered skin models alone, an effect that was dose-dependent and significantly different with the higher dose. The degree of contraction was compared with that of the untreated tissue-engineered skin model (100%). By day 14, the models with 10,000 ADSC contracted 77% ± 15%, whereas the models with 50,000 ADSC contracted 50% ± 12% (Figure 6a,b).

## Discussion

The development of skin contractures following skin damage can negatively affect the mental health and physical abilities of those affected [6]. Autologous fat grafting is currently used to restore volume beneath scarred skin and has demonstrated its ability to improve skin pliability and colour [13]; however, its effect on skin contraction remains poorly understood.

In this study, we demonstrated that adipose tissue significantly reduced skin contraction in a tissue-engineered skin model. This tissue-engineered skin contraction model was able to objectively demonstrate measurable changes in the skin following adipose treatment, which is very hard to determine in highly diverse patient scars. Using this model, comprising primary human keratinocytes and fibroblasts (isolated and used fresh) on a mature dermal scaffold (sterilized and preserved with glycerol [18]), we were able to study the effect of fresh human adipose tissue and ADSC on skin contraction.

The untreated skin model contracted over 14 days in culture, with the majority of contraction occurring over the initial 7 days, after which the model remained static and viable for up to 28 days in culture [17]. The addition of adipose tissue beneath the dermis led to a significant reduction in contraction, strongly supporting the use of autologous fat grafting to prevent skin contraction.

Different amounts of adipose tissue were administered to the model to test the effect of the adipose tissue dose on skin contraction. The three doses tested in this study were all able to prevent skin contraction to a similar degree (approximately 40% of the control skin), suggesting that the therapeutic effect of adipose tissue was not improved by adding more tissue. The doses tested here were equivalent to 70–300 µL per cm^2^ of skin, which was considerably lower than the doses used clinically (for example 1 mL adipose tissue per cm^2^ of skin was used by [19]). Greater quantities of adipose tissue could not be added to our model due to restrictions on the space available, which is a limitation; however, these limitations also exist in clinical practice, where the quantity of fat that can be administered to an area of skin is confined by tissue structure and anatomy.

In order to improve the effectiveness of AFG for different applications, alternative harvesting, processing and delivery techniques have been previously investigated which are reviewed here [20]. Processing methods include emulsification to lyse adipocytes [21] and condensation to remove tissue bulk and increase the concentration of stromal vascular fraction (SVF) [22]. In this study, emulsified fat (equivalent to nanofat 2.0 [21]) was tested. Adipose tissue was passed between Luer-Lok syringes to lyse the adipocytes and generate emulsified fat. Emulsified fat had the same effect on contraction as unprocessed adipose tissue, which may indicate that live adipocytes did not contribute to the effects on contraction or that adipocytes were not lysed as expected [23].

Adipose tissue was further processed to produce lipocondensate, which has been described by others as SVF-gel [22] or cell-enriched lipid tissue [24]. During this process, free lipids and the aqueous component were removed to condense the SVF component of adipose tissue. Processing adipose tissue to lipocondensate did not affect the tissue’s ability to inhibit contraction. This may indicate that the degree of contraction that can be inhibited is maximal with the dose of cells in unprocessed adipose tissue or this could be due to changes in cell behaviour and survival following processing.

Many of the regenerative properties of adipose tissue have been ascribed to ADSC found within the stromal vascular fraction of adipose tissue [25]. To test whether ADSC were responsible for the prevention of contraction observed here, cultured ADSC were added beneath the tissue-engineered skin model. When a higher dose of cells (50,000) was administered to the skin model, ADSC were able to prevent approximately 50% of contraction, similar to the prevention seen after adipose tissue administration. This effect appeared to be dose-dependent, as the lower dose of ADSC (10,000) only reduced contraction by 23%.

The lower dose of ADSC tested in this study (10,000) was selected to be comparable to the concentration of ADSC in 100 µL of unprocessed fat (estimated to be between 5,000 and 20,000 ADSC per 100 µL [26]). This lower dose of cultured ADSC was unable to prevent the degree of contraction to the same level as 100 µL adipose tissue treatment, suggesting that it is not solely the ADSC within the adipose tissue, which are responsible for the effects on contraction. Higginbotham et al., demonstrated that factors secreted from adipose tissue and cultured ADSC have different effects on myofibroblasts [14], further demonstrating the importance of studying adipose tissue as a whole to understand the function of AFG.

In this model, contraction was dramatically reduced to a level that would be clinically meaningful if the same can be achieved in clinical practice; however, contraction could not be blocked completely with adipose tissue or ADSC treatment. This may be the limit of prevention, or may indicate that repeated applications or higher doses are required. The model presented here represents the early stages of wound healing, when it may not be appropriate to completely block contraction, as contraction is a critical feature of wound closure and wound healing [3]. The data from this study suggest that AFG may be useful in preventing skin contraction and could, in theory, be used in combination with split-thickness skin grafts to reduce their contraction [10].

Wound healing is a multistage process involving numerous different cell types and biological processes. The tissue engineered model presented here most closely models the proliferative phase of wound healing. During this stage, keratinocytes proliferate to facilitate reepithelialisation, myofibroblasts promote scar formation and wounds can contract. This study focused solely on the effect of adipose tissue on skin contraction, a major clinical problem following skin grafting. Skin contraction has always proved difficult to study clinically and the contraction of human skin cannot easily be reproduced in loose skinned animals such as rats, mice or rabbits. The strength of this study is that the model demonstrated skin contraction and we were able to use it to answer the question of whether adipose tissue was able to prevent this contraction.

While this study focused solely on the effect of adipose tissue on skin contraction it has potential as a tool to look at other features of wound healing such as the rate of reepithelialisation and markers of scar formation, in the future. Our previous study demonstrated that paracrine factors from adipose tissue were able to reduce the proportion of fibroblasts expressing alpha smooth muscle actin, indicating a reduction in myofibroblast differentiation [14]. This reduction of myofibroblasts may also occur in the tissue engineered model presented here; however, it was not possible to draw conclusions from immunostaining of alpha smooth muscle actin due to the low density of fibroblasts visible in each histological section.

Adipose tissue was added to the model when the epithelium was a single layer, and the epithelium stratified during the period of adipose treatment [17]. In our previous study, we demonstrated that tissue-engineered skin treated with adipose tissue has a more consistent epidermal thickness and a more physiologically relevant dermal-epidermal junction [17]. This suggests that treating the skin with adipose tissue as it regenerates supports the development of a healthy dermal-epidermal junction, including rete ridges. Rete ridges are important for skin function and structure [27] but these features rarely reform following full-thickness wound healing [28] making this an interesting area worthy of further investigation.

In conclusion, the results of this study support the use of AFG to prevent skin contraction and demonstrates that this effect is in part, but not solely, a result of ADSC found within adipose tissue. Our observations indicate that reductions in contraction were possible with subclinical doses of adipose tissue and future work is needed to evaluate the impact of further increases in dose for both ADSCs and adipose tissue. Further studies are also required to elucidate the mechanisms responsible for the changes observed and to develop and optimize better treatments for patients living with contractures and contracted scars, where current treatment options are inadequate. We hope that the *in vitro* model presented here can be used in the future to improve our understanding of skin contraction and to support the objective quantification and evaluation of novel therapies aimed at preventing and treating contraction and skin contractures.

## Materials and methods

### Ethics

Waste hypodermal adipose tissue and skin were collected from routine surgical procedures in accordance with the Helsinki Declaration, with written informed consent, following protocols approved by the NHS Research Ethics Committee (refs: 15/YH/0177 & 21/NE/0115). All tissues were obtained and used on an anonymized basis from the Sheffield Teaching Hospitals NHS Trust, Directorate of Plastic, Reconstructive Hand and Burns Surgery, or Spire Claremont Hospital, Sheffield. The samples were stored at room temperature for up to 24 h after excision before processing.

### Cell media

Keratinocytes were cultured in Green’s media as described in [17]. Fibroblasts were cultured in DMEM (Sigma-Aldrich, #D6546) supplemented with 10% foetal calf serum (FCS, PAN-BioTech #P40–37500), 2 × 10^−3^ mol/L L-glutamine (Fisher Scientific #10691233), 0.625 mg/mL amphotericin B (Merck, #A2942) and 100 IU/mL penicillin, and 100 mg/mL streptomycin (Sigma-Aldrich, #P4333). ADSC were cultured in MesenPRO Reduced Serum (RS) (ThermoFisher Scientific #12746012) medium containing Growth Supplement (provided with MesenPRO RS media), 100 U/mL penicillin, 100 mg/mL streptomycin, 2 × 10^−3^ mol/L L-glutamine, and 0.5 mg/mL amphotericin B. All cells were sub-cultured with Trypsin-EDTA solution (5 mg/mL trypsin and 2 mg/mL EDTA) (Sigma-Aldrich, #T2934).

### Skin cell isolation and culture

Keratinocytes and fibroblasts were isolated from human skin according to the methods described previously [29]. Keratinocytes were detached (0.1% w/v Difco trypsin; BD Biosciences #215250) from split-thickness skin grafts, scraped from the surface of the dermis, and cultured in Green’s medium with an irradiated mouse fibroblast i3T3 feeder layer. Keratinocytes were isolated from five donors and used between passages 1 and 4.

Fibroblasts were isolated from the remaining dermal tissue (0.05% collagenase A (Merck, #10103578001) at 37°C for 24 hours) and cultured in DMEM supplemented as described above. Fibroblasts were isolated from four donors and used between passages 4 and 9.

### ADSC isolation and culture

Collagenase I (0.1% (w/v)) (Merck, # SCR103) in Hanks Balanced Salt Solution (HBSS, Thermo Fisher, #24020091) containing 0.1% (w/v) BSA (Merck, #A7030)) was mixed 1:1 (v/v) with minced adipose tissue and incubated at 37°C for 40 min to isolate ADSC. ADSC (Figure 1d) were cultured in supplemented MesenPRO medium, isolated from two donors, and used between passages two and six.

### Preparation of acellular decellularised dermis

Cadaveric donor skin (ETB-BISLIFE, The Netherlands) was processed to produce decellularised dermis (DED) (Figure 2a) as previously described [30]. Briefly, glycerol-preserved cadaveric skin was washed with phosphate-buffered saline (PBS) to remove glycerol, soaked in 1 M sodium chloride at 37°C until the epidermis could be separated from the dermal surface and washed again in PBS. DED from a single donor was used for each biological repeat, wherever possible, cut from the same skin sheet.

### Adipose tissue processing

#### Minced adipose tissue

Hypodermal adipose tissue was obtained from three donors. Large pieces of connective tissue and blood vessels were removed, and the tissue was minced thoroughly until tissue was the consistency of lipoaspirate (Figure 1a). Referred to as ‘adipose tissue’ this formulation was used in all experiments, unless stated otherwise.

#### Emulsified adipose tissue

Emulsified adipose tissue (also called Nanofat 2.0) was produced as previously described [21]. Minced adipose tissue was emulsified by passing the tissue 30 times between two 10 cc syringes through a Luer-Lock connector (Figure 1b).

#### Lipocondensate

Emulsified adipose tissue was processed to generate lipocondensate, according to a previously described protocol [22]. Briefly, following emulsification, the adipose tissue was filtered through a 500 µm filter (pluriStrainer, Cambridge Bioscience, #43 -50,500-03) to remove connective tissue, and centrifuged at 2000 × g for 3 min. Multiple layers were formed by centrifugation, and the lipocondensate layer was removed from between the oil layer and the red aqueous layer (Figure 1c).

## Production of tissue engineered skin models

The DED was cut into 15 × 15 mm squares and placed into a 6 well plate with the papillary surface facing upwards. A stainless-steel ring (internal diameter 1 cm) was pushed onto the DED to form a tight seal. Green’s media was placed into the well around the stainless-steel ring to maintain hydration. Inside the ring 3 × 10^5^ keratinocytes and 1 × 10^5^ fibroblasts were seeded per model in 500 µL of Green’s media. The cells and DED were cultured submerged for 24 h with the ring in place and for a further 24 h with the ring removed. To aid in the analysis, the cell-seeded area was cut out to produce circular models with a diameter of 1 cm. The cut out models were placed on a stainless steel grid to generate an air – liquid interface (ALI), which is herein described as day 0. Models were cultured in standard tissue culture conditions (humidified incubator at 37°C with 5% CO_2_) in Green’s medium at an air-liquid interface for 14 days, and the medium was replenished every 2–3 days.

## Addition of adipose tissue or ADSC to tissue engineered skin models

Adipose tissue or ADSC were added to the tissue-engineered skin models at day 0, as the models were raised to ALI and before the epidermis had stratified. Keratinocytes, fibroblasts, ADSC, and adipose tissue were not from matched donors in any of the models. To avoid tissue or cells escaping through the holes in the stainless steel grid a PTFE membrane (0.45 µm, 25 mm diameter; Merck-Millipore #JHWP02500) was placed above the stainless-steel grid (Figure 1e). Adipose tissue was added underneath each model using a syringe or positive displacement pipette. ADSC were added to the PTFE membrane before the tissue-engineered skin was placed on the top.

## Image capture and image analysis

Photographs were taken using a digital microscope (Celestron) at regular intervals between days 1 and 14 of culture at ALI, to measure the area of the models over time. The area of each model was calculated using ImageJ software (version 1.53c) [31].

## Histological analysis

Tissue-engineered skin models were fixed with 10% phosphate-buffered formaldehyde and processed for conventional histological examination using haematoxylin and eosin (H&E) staining. Bright-field images of stained slides were taken using an upright compound microscope (Olympus CX43) and Euromex HD-Ultra camera (both supplied by Best Scientific).

## Data analysis and statistics

Technical and biological replicates were performed to account for patient variations (see the legend for details). The small number of models that failed to form a stratified epithelium when assessed by histology were excluded from the analysis.

The surface area of each model was measured at each time point and the change in area was calculated respective to the same model at time 0. The degree of contraction is presented relative to the contraction of the control model; DED seeded with fibroblasts and keratinocytes but not treated with adipose tissue or ADSC. The control model was reported as 100% contraction and models cultured from the same cells and DED but treated with adipose tissue or ADSC were compared against this to control for donor variation. The DED control contained no cells and acted as the negative control. DED controls were not treated with adipose tissue or ADSC.

Data are presented as the mean ± standard deviation (S.D). Two-way Analysis of Variance (ANOVA) with Tukey multiple comparison was used to compare the effect of different treatments with control models. A p-value <0.05 was considered statistically different.

## Data Availability

For the purpose of open access, the author has applied a Creative Commons Attribution (CC BY) licence to any Author Accepted Manuscript version arising. The data that support the findings of this study are openly available in the University of Sheffield’s data repository ORDA. Access: DOI:10.15131/shef.data.26485597.

## Abbreviations

ADSC: Adipose Derived Stromal Cells
AFG: Autologous Fat Grafting
ALI: Air Liquid Interface
BSA: Bovine Serum Albumin
DED: Decellularised Dermis
DMEM: Dulbecco’s Modified Eagle Medium
EDTA: Ethylenediaminetetraacetic acid
EGF: Epidermal Growth Factor
FCS: Fetal Calf Serum
H&E: Haematoxylin and Eosin
HBSS: Hank’s Buffered Saline Solution
NHS: National Health Service
PBS: Phosphate Buffered Saline
PTFE: Polytetrafluoroethylene
RS: Reduced Serum
SD: Standard Deviation
